# Contextual barriers to implementation in primary care: an ethnographic study of a programme to improve chronic kidney disease care

**DOI:** 10.1093/fampra/cmw049

**Published:** 2016-06-13

**Authors:** Natalie Armstrong, Georgia Herbert, Liz Brewster

**Affiliations:** ^a^Department of Health Sciences, University of Leicester, Leicester, UK,; ^b^NIHR Biomedical Research Unit in Nutrition, Diet and Lifestyle at the University Hospitals Bristol NHS Foundation Trust and the University of Bristol, Bristol, UK and; ^c^Lancaster Medical School, Faculty of Health and Medicine, Lancaster University, Lancaster, UK

**Keywords:** Context, general practice, Great Britain, implementation, primary health care, qualitative research.

## Abstract

**Background.:**

Context is important in implementation—we know that what works in one setting may not work in the same way elsewhere. Primary care has been described as a unique context both in relation to the care delivered and efforts to carry out research and implementation of new evidence.

**Objective.:**

To explore some of the distinctive features of the primary care environment that may influence implementation.

**Methods.:**

We conducted an ethnographic study involving observations, interviews and documentary analysis of the ENABLE-CKD project, which involved general practices implementing a chronic kidney disease care bundle and offering self-management support tools to patients. Analysis was based on the constant comparative method.

**Results.:**

Four elements of the primary care environment emerged as important influences on the extent to which implementation was successful. First, the nature of delivering care in this setting meant that prioritizing one condition over others was problematic. Second, the lack of alignment with financial and other incentives affected engagement. Third, the project team lacked mechanisms through which engagement could be mandated. Fourth, working relationships within practices impacted on engagement.

**Conclusions.:**

Those seeking to implement interventions in primary care need to consider the particular context if they are to secure successful implementation. We suggest that there are particular kinds of interventions, which may be best suited to the primary care context.

## Introduction

Ensuring high quality is a priority for primary care, but UK primary care has traditionally demonstrated high variability in care quality (1,2). This has often been addressed through structural or system-level mechanisms such as the Quality and Outcomes Framework (QOF) (3). Improvement interventions using recognized methodologies to implement change and improve care quality have seen much less penetration (4).

Primary care has been described as a unique context (5), and the evidence-to-practice gap for complex interventions in this setting is currently receiving attention (6). With some exceptions, implementation in primary care tends to be understudied compared with other settings such as hospital care, despite evidence of the importance of contextual modifiers (4,7). The context in which implementation takes place is important, and better understanding of how context influences implementation can help explain why the same intervention may have a significant impact in one setting, but ‘fail’ when attempts to implement it elsewhere are made (8).

Previous research on implementation in primary care has shown that staff may lack experience of using recognized improvement approaches (9), and there may be limitations in the capacities and capabilities of the workforce to undertake systematic improvement (4). Although other factors—such as stakeholder motivation and resources, external motivators and opportunities for change (10)—have a role to play, this perceived skills gap may also be important. Improvement efforts tend to be disease focused or pathway specific, and changes are not always sustained or spread across practices. Using ‘practice facilitators’ to support change has been identified as a possible solution, but does not appear to have a longer-term effect on culture (9). Tailoring the intervention to the practice may have positive outcomes, though this may be a ‘messy and iterative process’ (11) not necessarily appropriate for large-scale roll-out.

A better understanding of how the primary care context may influence attempts to improve care quality is vital if improvement efforts in this setting are to succeed (4). This paper focuses on a primary care-based improvement project seeking to improve the care of patients with chronic kidney disease (CKD). We examine how aspects of the primary care setting influenced implementation, paying particular attention to the challenges it posed.

## Methods

We used a multi-method case study approach to look at one improvement project in UK primary care. The project, *Enhancing Care and Saving Lives of People with Chronic Kidney Disease* (ENABLE-CKD), was hosted by Kidney Research, UK (a charity) and funded by the Health Foundation (an independent charitable foundation) as part of a programme of 11 projects seeking to close the gap between best evidence and current practice (12).

CKD is estimated to affect 5–10% of the population and is associated with cardiovascular mortality and morbidity (13). Performance in relation to CKD management in UK primary care has been linked to financial reward though the QOF (Box 1).

Box 1. Chronic kidney disease (CKD)-related indicators included in the Quality and Outcomes Framework (QOF) at the time of the intervention*

- The practice can produce a register of patients aged 18 years and over with CKD (US National Kidney Foundation: Stage 3 to 5 CKD).
- The percentage of patients on the CKD register whose notes have a record of blood pressure in the preceding 15 months.
- The percentage of patients on the CKD register in whom the last blood pressure reading, measured in the preceding 15 months, is 140/85 or less.
- The percentage of patients on the CKD register with hypertension and proteinuria who are treated with an angiotensin-converting enzyme inhibitor (ACE inhibitor) or angiotensin receptor blocker (ARB).
- The percentage of patients on the CKD register whose notes have a record of a urine albumin:creatinine ratio (or protein: creatinine ratio) test in the preceding 15 months.

*Since this study was undertaken, the QOF CKD-related indicators have been revised over time and the majority now retired. All that remains for the 2015/16 year is the indicator related to establishing and maintaining a register.

The gap identified by ENABLE-CKD was between contemporary National Institute for Health and Care Excellence (NICE) guidance on best practice for CKD management in primary care and current practice, evidenced by apparent problems in recorded prevalence rates in CKD registers and exception reporting (13).

The ENABLE-CKD project team sought improvement by trying to establish consistent implementation of NICE guidance; building confidence through increased understanding of CKD; and facilitating collaborative self-management with CKD patients. To achieve these aims, they (i) advised general practices on how to improve the quality of their CKD registers, or worked with their baseline data to develop a register if one did not exist (a QOF indicator—Box 1); (ii) provided training sessions and additional supporting materials to practices, which aimed to build the knowledge and skills of staff on optimal CKD management and patient self-management; and (iii) encouraged general practices to use a care bundle approach. The elements of the care bundle are shown in Box 2; the protocol did not prescribe a specific setting for bundle application but suggested settings included a dedicated CKD clinic, a generic long-term conditions clinic or *ad hoc* delivery (14).

**Box 2. Items comprising the enhancing care and saving lives of people with chronic kidney disease (ENABLE-CKD) care bundle**

A. Ask the patient whether they want to take part in a self-management programme.
B. Measure and document proteinuria and prescribe appropriate medication (ACEi/ARB) if proteinuria present.
C. Document Blood Pressure (BP) and treat if above NICE (2008)/SIGN targets.
D. Document cardiovascular (CV) risk using an appropriate CV risk calculator e.g. QRisk2 (www.qrisk.org).

Following the training session, practices were asked to supply monthly data on their bundle implementation rates and to participate in progress review teleconferences. Training was completed in 29 practices and 26 returned baseline data. Ten practices dropped out after the training. Of the remaining 19, 13 submitted at least six sets of monthly practice-level bundle implementation data. The other six practices continued to apply the bundle but did not submit the required implementation data. The ENABLE-CKD team’s final analysis of bundle implementation in the 13 practices submitting the required data showed this ranged from <20% (five practices) to ≥50% (three practices) of CKD-registered patients (14). By the end of the project, 1313 patients had used the care bundle overall.

### Evaluation data collection and analysis

Our study design was a case study of ENABLE-CKD. We completed 16.5 days’ non-participant observation focusing on the project team’s activities, including team meetings, practice training sessions and workshop events. We also interviewed 14 members of the ENABLE-CKD team (including two external consultants) and 24 staff at a purposive sample of five participating practices (7 GPs, 9 nurses, 4 practice managers, 1 pharmacist, 1 self-management facilitator, 1 administrator and 1 IT support staff). Our data collection thus covered the ‘blunt end’ of project management through to the ‘sharp end’ where practitioners implemented change.

Practices were purposively sampled on the basis of geographical area (urban and rural) and practice size in terms of number of GPs (*n* = 3 to *n* = 10+). All practices approached were willing to be involved. We did not select practices on the basis of engagement with the intervention (i.e. submitting data to the ENABLE-CKD team) as this was not yet known. No additional incentive was offered to participate in the evaluation and only one person approached for interview declined to participate.

Observers made written notes during observations, using a sensitizing framework to ensure notes focused on the implementation of improvement work. Team debriefs, in which observers came together to discuss emerging themes, were then conducted, audio-recorded and transcribed. Interviews were also audio-recorded and transcribed. Relevant project documents were analysed, including project plans, reports and training materials.

Data analysis was based on the constant comparative method (15). Through comparison across interview and observation transcripts, initial open codes were organized into thematic categories, which provided a framework for processing all data using QSR NVivo software. All data collection and analysis was completed by members of the independent evaluation team (rather than the ENABLE-CKD team).

## Findings

Four distinctive features of the primary care context emerged as important influences on implementation: prioritization within general practice; the relative lack of financial or other incentives to encourage participation; the lack of mechanisms through which to mandate engagement; and working relationships within practices.

### Prioritization within general practice

General practice is, by its very nature, concerned with the delivery of general rather than specialist health care—meaning that practices and the staff working within them typically have to make decisions about what activities to prioritize. In contrast, many improvement interventions, as was the case with ENABLE-CKD, tend to be focused on specific conditions and/or processes. Tensions between the wide-ranging activities of primary care and the specifics of managing CKD recurred throughout the project. Problems arose, and improvement efforts sometimes stalled, because practice staff’s time was divided among many competing demands.

I think it’s just pressure of time really—splitting our energies and our focus across such a broad area of clinical problems. (GP 2)

Some conditions (e.g. diabetes or heart disease) fare well in this prioritization, but others do not. One of the reasons that ENABLE-CKD encountered problems getting practice staff to prioritize the work they were asking them to do was related to the legitimacy of CKD as a clinical priority.

I had somebody sitting in that chair yesterday—I was more concerned about their liver and he said “oh, how are the kidneys?” and they were fine, he’s got really good EGFR. He could live out his life without any problems but he’s now spending every day worrying about his kidneys. It’s medicalising something in the patient’s mind and exaggerating the impact of it on their lives. (GP 2)

These issues combined to mean that busy practice staff were not always willing and/or able to prioritize CKD-related activity over other things.

### Financial incentives

Practice staff talked about ‘running a business’, with a focus on budgeting. Fiscal pressures resonated and priority was given to activities providing the most financial gain. Staff were especially driven to align their activities with the QOF.

The QOF takes preference over pretty much everything, because that’s the big earner for the practice, that’s what keeps the practice running. (Practice manager 1)

[QOF has] a financial implication for funding of how the practice runs, funding wages, funding lighting, everything fundamental about the practice. (GP 1)

Because of the financial consequences, meeting QOF targets was often a motivator for initial engagement with ENABLE-CKD; practices were attracted by the specialist training and expertize offered by ENABLE-CKD.

GPs’ lives revolve around QOF. So if something’s not in QOF then it gets pushed to the back, and it’s not a focus. Bringing [CKD] into QOF certainly made us look a bit harder at what we were doing. (Practice manager 1)

We knew that CKD was an up-and-coming area of clinical practice that was being talked about in all of the medical press and we realised that we weren’t compliant with the QOF. We’re going to have to make changes in order to comply with the QOF expectations and it was an opportunity to get our act together really. (GP 2)

However, while there was overlap between the QOF indicators and the care bundle proposed, there were important differences. First, self-management for CKD was not included within the QOF indicators, meaning that engagement with this aspect went beyond that for which practices could expect to be financially rewarded. Second, the blood pressure target included in the care bundle was taken from NICE guidance at the time (120–139/90) rather than from QOF itself (140/85) and was thus more challenging to achieve.

Once practices had obtained all the QOF points available for CKD, they had little incentive to go further and meet the other objectives of ENABLE-CKD such as the self-management element or the tighter blood pressure control.

Whilst maybe our project might have helped with their CKD QOF, it will have taken away attention from all their other QOFs, so we had to work within that and to us, you know, it’s all about kidneys...but it’s obviously not to them. (Project team member 2)

This focus on the bottom line presented a major challenge in securing engagement. Practice staff often balanced the desire to improve care with the financial implications of implementation, and thus some practices showed little desire to engage fully with the project unless it was financially beneficial or at least cost neutral. Some practices presented ENABLE-CKD with full costings (e.g. backfill for staff attending training, intervention set-up expenses) and requested reimbursement. ENABLE-CKD, despite some reservations and having not initially planned for this, secured additional funding to cover these costs as they felt being able to offer funding was necessary to ensure credibility in an environment, where this appeared to be the norm.

### Lack of mechanisms to mandate engagement

ENABLE-CKD were not prepared for how autonomously general practices functioned; each practice essentially operated in isolation as a small business and was not part of a wider accountability structure such as a hospital trust. Identifying ‘sticks’ to motivate practices to engage was difficult.

ENABLE-CKD are using lots of soft tactics, dangling lots of carrots in front of the practices, being collaborative, nice, but says that sometimes this isn’t enough: are there harder edges? Are there sticks as well as carrots? [Project team member] says, GP practices would just say “stuff it, go away then, we won’t work with you” and [project team member] says “it’s a different ball game” and [project team member] says “they are very autonomous.” (Observation de-brief)

As a result, ENABLE-CKD relied primarily upon softer tactics, such as appealing to clinicians’ desire to follow best practice. This reliance on soft tactics had several consequences. First, it was difficult to generate momentum, not least because there were competing ‘hard edges’ already in place that played a significant role in guiding activities (such as the QOF).

QOF says you don’t need to do it for everybody all the time. That’s the basic problem [...] You can miss things out and still get QOF points and hence get remuneration. It doesn’t ask you to do everything it just says that if you do some things well but other things not so well, we’ll still give you some points. (GP 3)

Second, while using soft tactics might attract enthusiasts who were already motivated to tackle CKD, it could do little to engage those who were more sceptical. Third, it was time and labour intensive, relying on constant communication to sustain engagement.

Fourth, using soft tactics, the project team inevitably occupied a less powerful position in encouraging continued engagement, which resulted in some practices taking time to begin implementation of the care bundle, if indeed they did so at all.

[Project team member] says “often moving them [the participating sites] forward, it’s difficult to do…” And she said that she feels like a nagging woman. (Observation de-brief)

The ENABLE-CKD team felt that practice engagement was stronger when they had buy-in from multiple stakeholders, including GP partners, practice managers and the nurses, who ultimately delivered most of the intervention. Although the relative autonomy of general practices caused difficulties, it did offer some advantages; an enthusiastic practice that wanted to commit to improvement in this area had the independence to do so. Control over resources (such as facilities and staff) tended to lie within the practice itself and therefore motivated practices could quickly and easily implement change.

### Staff working relationships

Working relationships within practices were found to be different to secondary care (the context with which ENABLE-CKD was more familiar), and this impacted on engagement and implementation. The employer/employee relationship within primary care (i.e. that GP partners employ all other practice staff) created important power dynamics. Even though nurses would typically be responsible for implementing the intervention and many were willing to do so, GPs and practice managers tended to have the final say over whether they did so or spent their time on other things. Thus, if GPs and managers were not engaged and did not regard this work as a priority or good use of time, nurses could not push things forward on their own, even if they were enthusiastic. Without the necessary gatekeepers to authorize activity, little could be accomplished.

I’m not sure how it’s going to be instigated at the moment, I think that’s obviously going to be decided higher up. (Practice nurse 4)

SO WHAT WERE YOU HOPING TO GET OUT OF THE TRAINING?

I suppose more of an awareness, it’s not something I know a great deal about, just really to... I think I am going to be used in some sort of role with the study, but I’m not sure, so I was just asked to come down and participate really. (Practice nurse 3)

In training sessions, the ENABLE-CKD team was conscious of the potential for nurses to feel excluded and tried to ensure that all groups were engaged in discussion. Two project team members had a nursing background and were sensitive to the potential for nurses’ voices to be marginalized; however, even they appeared surprised at the stark contrast between the secondary and primary care context.

The hierarchy is much greater in primary care than in secondary care. The nurses hardly say anything; the GPs are in control, because they employ them. That’s the difference with secondary care. (Project team member 1)

## Discussion

Effective implementation requires sensitivity to context and there are some important features of the primary care environment that need to be taken into account. This case study illustrates how the particular context of primary care can pose challenges for implementation. First, the need to prioritize CKD over many other areas of activity affected engagement. The value proposition of ENABLE-CKD was not always clear. CKD was only one small concern among many priorities; it was a specialty interest not part of the mainstream workload. This supports previous suggestions that measuring indicators that transfer across different chronic conditions and co-morbidities meet with greater success than those focused on a specific condition (16).

Second, the nature of general practices as small businesses influenced motivation to implement change. While the issue of financial incentives driving clinical activity is, of course, not unique to either primary care or CKD, the ways in which they played out in this case are of interest. As private businesses with revenue streams linked to specific targets, practices sometimes struggled to accept that CKD management required action over and above that required to generate QOF points. Practices were accustomed to being paid for participation in ‘non-core’ activity and some sought payment for participation here. Although the evidence about the effectiveness of pay-for-performance on outcomes is mixed (17), ENABLE-CKD’s experience suggests this needs to be considered.

Third, ENABLE-CKD struggled to identify effective ways of mandating engagement; it was easy for practices to opt out from some or all elements. While clinicians’ motivation to deliver high-quality care can help secure participation, relying on this ‘carrot’ may not always be enough—‘sticks’ may be needed to encourage change more effectively (18). However, even when available these do not also facilitate engagement. External mandates and performance management may provide an initial motivation for action (19), but enforcing participation in an initiative may have unintended consequences (20).

Risks include the focus being placed on a particular target, with other important priorities being ignored—known as effort substitution—and manipulation or ‘gaming’ of the data for gain. Using ‘sticks’ may also adversely affect relationships, with staff feeling that their clinical autonomy and judgement is undermined by enforcement (21).

Finally, working relationships and the locus of power was significant. While nurses typically were the implementers here, GPs and practice managers needed to authorize this. The status of nurses as employees of the practice could problematize effective engagement. Concerns about hierarchy have already been identified as hampering multidisciplinary work (22).

When looking at implementation of complex interventions in primary care, context has often been ignored. A systematic review of reviews identified professional, organizational, financial and regulatory strategies to implement change (6). The review concluded that there is little evidence of effectiveness because these strategies fail to take into account the role of the context of implementation. Our findings support this conclusion, with the degree of fit between the intervention and the context in which it was being implemented as the most influential interrelationship.

Our findings suggest that the intervention ENABLE-CKD sought to implement was perhaps not best suited to the primary care context: it was not a priority for many; did not always fit well with external motivators/incentives; could not be mandated; and had not sufficiently taken into account the relationships between those who needed to be involved in implementation. The same review looked at features of effective implementation and identified audit and feedback, educational strategies and financial incentives as most useful (6).

However, they need to be tailored to the primary care setting and particular purpose, and financial incentives need to be large to be successful. ENABLE-CKD did use educational sessions and these were positively reviewed by participants; they tried to use audit and feedback but without ‘teeth’ this largely fell flat, and while they did ultimately provide some funding, financial incentives were not initially built in.

The issues identified as problematic in this case study need not always be so—they could be alleviated through more optimal alignment of intervention and context. For example, the ‘payment culture’ was experienced as a challenge, but if factored in early on could be an opportunity to promote engagement through identification of a strong business case. Also, despite doubts about whether financial incentives are adequately aligned to maximize health gains, evidence shows QOF has changed clinicians’ behaviour (17); better alignment with the current incentive system could have been beneficial. Finally, for ENABLE-CKD, practice autonomy largely worked against implementation efforts. As autonomous organizations, practices approached change with caution. However, when clinicians were more willing to engage, this could be extremely valuable; being free of bureaucracies and hierarchies could lead to quick and efficient implementation.

This paper presents data from one, condition-focused, improvement project, although the purposively sampled data come from several locations and sources. Conceptual transferability, not statistical generalizability, was the priority. While ENABLE-CKD experienced some significant challenges, not all of which they were able to tackle successfully, this evaluation provides important insights into the nature of these challenges and how they influenced implementation, and in particular adds to the evidence focused on the role that context plays in implementation in primary care, identified as a current gap in research knowledge (6).

Understanding the context in which you are trying to implement change is vital, and the specific characteristics of primary care are no exception (4). In the case of ENABLE-CKD, project team members were not themselves from a primary care background and so, as evidenced here, they sometimes struggled to navigate this unfamiliar terrain. From the outset, the project team struggled to secure consistent, meaningful input from primary care professionals at the project level, despite their best efforts to do so. Teams require members with ‘insider knowledge’ to highlight potential problems, identify strategies likely to be effective in any setting and maximize the likelihood of sustainability (23).

Improvement is a priority in all health care contexts, and this study identifies some of the factors that may influence its implementation in primary care. A lack of awareness of the specific facets of the environment may affect outcomes, as shown in this study. Further work needs to determine to what extent the challenges experienced by the ENABLE-CKD project are found in other cases. Future improvement work will need to be embedded in the context and culture of primary care in order to ensure success.

## Declaration

Funding: the Health Foundation (registered charity 286967) as part of its evaluation of the Closing the Gap through Clinical Communities programme. Liz Brewster’s contribution was supported by Mary Dixon-Woods’ Wellcome Trust Senior Investigator Award (WT097899).

Ethical approval: the Leicestershire, Northamptonshire & Rutland Research Ethics Committee 1(10/H0406/77).

Conflict of interest: none.
